# Correction: Genetic landscape of preterm birth due to cervical insufficiency: Comprehensive gene analysis and patient next-generation sequencing data interpretation

**DOI:** 10.1371/journal.pone.0249483

**Published:** 2021-03-25

**Authors:** The PLOS ONE Staff

There is an error in the author designations. Linda Gailite and Anna Miskova do not share first authorship of the work. Instead, Linda Gailite and Anna Miskova share senior authorship of the work. The publisher apologizes for the error.
