# Supplementary figures and images for: Genetic landscape of preterm birth due to cervical insufficiency: Comprehensive gene analysis and patient next-generation sequencing data interpretation
Source: PLoS One. 2020 Mar 26;15(3):e0230771. doi: 10.1371/journal.pone.0230771 (PMC7098624; doi:10.1371/journal.pone.0230771)

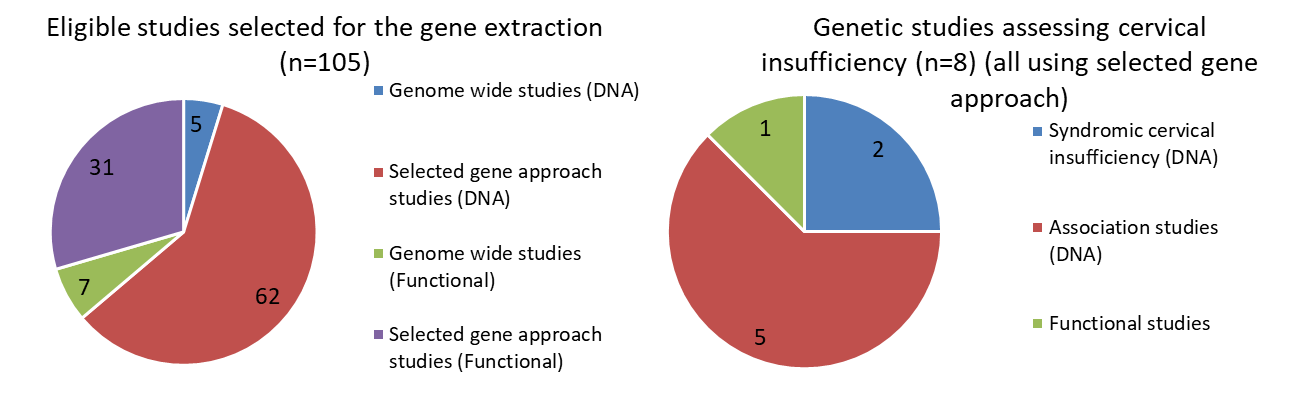

Supplement: S1 Fig — All studies included for the gene selection (left) and ones focusing primarily on cervical insufficiency (right). (TIF) [file pone.0230771.s010.tif]

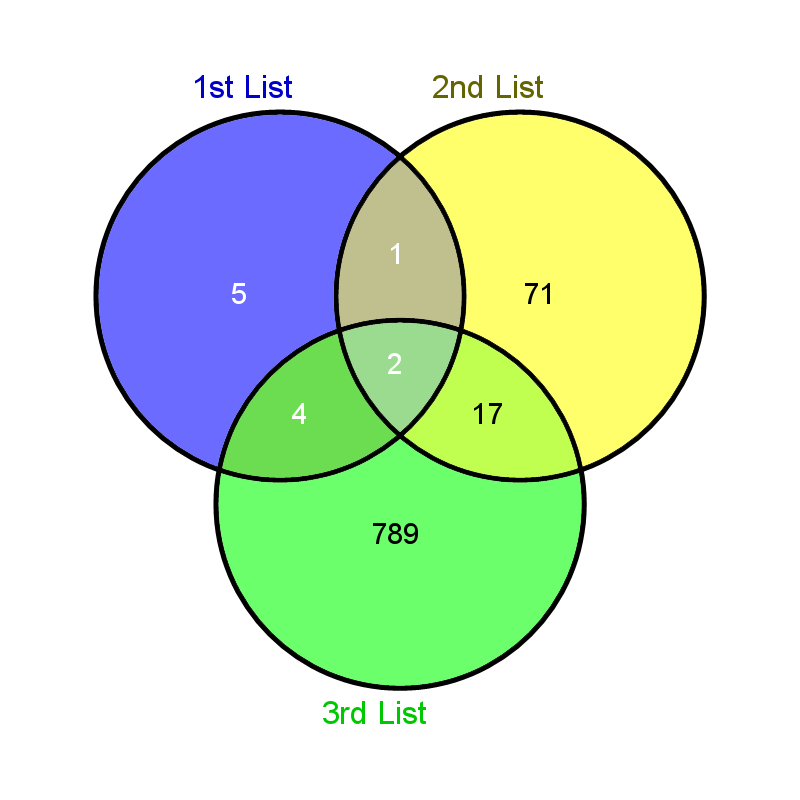

Supplement: S2 Fig — (TIF) [file pone.0230771.s011.tif]

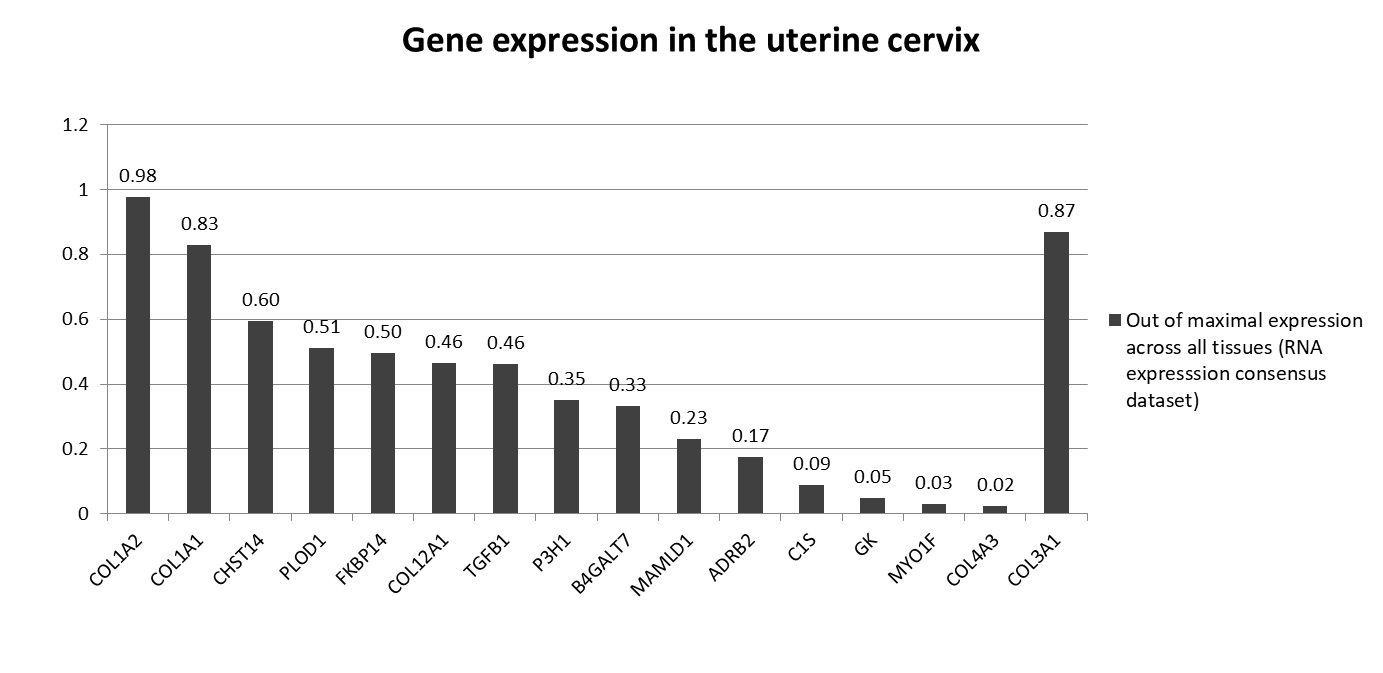

Supplement: S3 Fig — Genes found to be mutated in our patient cohort as shown through the 1st and 2nd gene list analysis. Data obtained through an RNA expression dataset available at https://www.proteinatlas.org. In our cohort, none of the rare or pathogenic variants were found in the COL3A1 gene; however, it is included as it is the only gene unequivocally linked to cervical insufficiency. (TIF) [file pone.0230771.s012.tif]
